# Supplementary material for: Evolutionary Analysis of Vertebrate KCNH Voltage-Gated Potassium Channels and Spatial Expression of kcnh Genes in Zebrafish Embryos
Source: J Dev Biol. 2026 Jul 13;14(3):32. doi: 10.3390/jdb14030032 (PMC13398235; doi:10.3390/jdb14030032)
Supplement: Supplementary file 1 [file jdb-14-00032-s001.zip › jdb-4315945-supplementary.pdf]

Table S1. Protein sequences used for phylogenetic analysis.

Common species name	Scientific Name	KCNH1	KCNH2	KCNH3	KCNH4	KCNH5	KCNH6	KCNH7	KCNH8
**Human**	Homo sapiens	NP_758872.1	NP_000229.1	NP_036416.1	NP_036417.1	NP_647479.2	NP_110406.1	NP_150375.2	NP_653234.2
**Mouse**	Mus musculus	NP_034730.1	NP_038597.2	NP_034731.3	NP_001074663.1	NP_766393.2	NP_001032801.1	NP_573470.2	NP_001026981.2
**Rat**	Rattus norvegicus	XP_017454422.1	NP_446401.1	NP_058804.1	NP_446082.2	NP_598294.1	XP_006247617.1	NP_571987.1	EDL83035.1
**Chicken**	Gallus gallus	XP_040522344.1	XP_015136636.3		XP_040509293.1	XP_040529709.1	XP_046789286.1	XP_046799783.1	XP_040520526.1
**Turkey**	Meleagris gallopavo	XP_031408030.1	XP_010722842.2			XP_010710334.1	XP_010722789.1		N/A
**Xenopus**	Xenopus tropicalis	KAE8600584.1	XP_031759591.1	XP_031753016.1	XP_002942364.2	XP_031746084.1	XP_012808358.2	XP_031749025.1	XP_031760363.1
**Eastern brown snake**	Pseudonaja textilis	XP_026551168.1		XP_026567024.1	XP_026566646.1	XP_026554170.1	XP_026573067.1	XP_026552928.1	XP_026564036.1
**Chinese softshell turtle**	Pelodiscus sinensis			XP_075758072.1	XP_075767445.1	XP_075782488.1	XP_075767334.1	XP_075789622.1	XP_006120233.2
**Coelacanth**	Latimeria chalumnae	XP_064411209.1	XP_014348120.1	XP_064408372.1	XP_064421690.1	XP_064421237.1	XP_014343047.1	XP_064420119.1	XP_064410955.1
**Elephant shark**	Callorhinchus milii	XP_007890670.1				XP_007902076.1		XP_007888033.2	XP_042188420.1
**Spotted gar**	Lepisosteus oculatus	XP_069035919.1	XP_006634281.2	XP_015217397.2	XP_015217710.2	XP_006632393.1	XP_015217397.2	XP_069052888.1	XP_069051169.1
**Nile tilapia**	Oreochromis niloticus	kcnh1a: XP_025753448.1	kcnh2b: XP_019218510.1	XP_005460711.1	kcnh4a:XP_005468906.1	kcnh5a: XP_003449970.1	kcnh6a: XP_005469007.1	kcnh7a: XP_013128067.1	XP_003448945.1
		kcnh1b: CAI5681500.1			kcnh4b:XP_005471361.1	kcnh5b: XP_003451242.1		kcnh7b: XP_025758714.1	

**Platyfish**	Xiphophorus maculatus	kcnh1a: XP_023183743.1	kcnh2b: XP_023182235.1	XP_014328923.1	kcnh4a: XP_023204390.1	kcnh5a: XP_014324695.1	kcnh6a: XP_014330674.1	kcnh7a: XP_023192648.1	XP_023200281.1
					kcnh4b: XP_023197359.1			kcnh7b: XP_014327628.1	
**Three-spined stickleback**	Gasterosteus aculeatus aculeatus	kcnh1a: XP_040033853.1	kcnh2a: ENSGACT00000053404.1	XP_040044960.1	kcnh4a: XP_040046504.1	kcnh5a: XP_040017369.1	kcnh6a: XP_040047636.1	kcnh7a: XP_040056596.1	XP_040045452.1
		kcnh1b: XP_040042776.1	kcnh2b: XP_040022853.1		kcnh4b: XP_040032699.1		kcnh6b: XP_040032088.1	kcnh7b: XP_040044756.1	
**Japanese medaka HdrR**	Oryzias latipes	kcnh1a: XP_020565084.1			kcnh4a: XP_020560803.1	kcnh5a: XP_011490256.1	kcnh6a: XP_011476138.1	kcnh7a: XP_011488040.1	XP_011479155.1
		kcnh1b: XP_023810050.1	kcnh2b: XP_011486965.1		kcnh4b: XP_004080285.1	kcnh5b: XP_023807694.1		kcnh7b: XP_020565953.1	
**Zebrafish**	Danio rerio	kcnh1a: XP_073783443.1	kcnh2a: XP_073775577.1	XP_001919436.3	kcnh4a: NP_001309362.1	kcnh5a: XP_073788639.1	kcnh6a: NP_998002.1	kcnh7a: XP_073769333.1	XP_073787754.1
		kcnh1b: XP_009294796.1	kcnh2b: XP_073797320.1		kcnh4b: XP_073774395.1	kcnh5b: XP_002664253.1	kcnh6b: XP_073774351.1	kcnh7b: XP_073809787.1	
**Mexican tetra**	Astyanax mexicanus	kcnh1a: XP_049320079.1	kcnh2a: XP_049323249.1	KAG9271189.1	kcnh4a: XP_022528303.2	kcnh5a: KAG9282826.1	kcnh6a: ENSAMXT00000012115.2	kcnh7a: XP_049340940.1	XP_049331969.1
		kcnh1b: XP_022525848.2	kcnh2b: XP_007256983.3		kcnh4b: ENSAMXT00000031902.1	kcnh5b: KAG9274900.1	kcnh6b:XP_049320999.1	kcnh7b: XP_049335102.1	
**Electric eel**	Electrophorus electricus	kcnh1a: XP_035388576.1	kcnh2a: XP_026861581.2	XP_026851708.2	kcnh4a: XP_035386833.1	kcnh5a: XP_026858766.2	kcnh6a: XP_035379849.1	kcnh7a: XP_026866052.2	XP_035385068.1
		kcnh1b: XP_026852208.2	kcnh2b: XP_026858752.1		kcnh4b: XP_026873473.2			kcnh7b: XP_035382667.1	

**Fugu**	Takifugu rubripes	kcnh1a: XP_029691127.1		XP_029696573.1	kcnh4a: XP_029691913.1	kcnh5a: XP_029705807.1	kcnh6a: XP_011602586	kcnh7a: XP_029703364.1	XP_003969315.2
			kcnh2b: XP_029698764.1		kcnh4b: XP_003961302.2	kcnh5b: XP_029688754.1/ ENSTRUT00000076157.1	kcnh6b: XP_029693733.1	kcnh7b: XP_029695709.1	
**Lancelet**	Branchiostoma lanceolatum	XP_066279784.1			CAH1249231.1	KCNH5	XP_066301818.1: kcnh6-like	CAH1238966.1	XP_066263673.1
**Tunicate**	Ciona intestinalis					XP_026694540.1	XP_026692366.1: Kcnh6		XP_009859607.1: kcnh8-like
**Common fruit fly**	Drosophila melanogaster	NP_001036275.1	NP_477009.1: eag-like	NP_001286814.1					
**Roundworm**	Caenorhabditis elegans	NP_001368562.1		NP_001368173.1					

Table S2. PCR primers for the zebrafish *kcnh* gene cloning.

Gene	Transcript ID	Primers	Sequence	PCR product size (bp)
kcnh1a	NM_001044931.1	Dr.kcnh1a-F	5'-GCCCCCTTGCCACCATGGCCGGGGGACGCAGAGGACTAG-3'	2880
		Dr.kcnh1a-R	5'-CGGCGCGCCCACCCTTAGGGAACATGTCCTCCTTGTCTGTGTCTGGC-3'	
kcnh1b	NM_001278814.1	Dr.kcnh1b-F	5'-GCCCCCTTGCCACCATGGCGGGGGGACGCAGAGGACTGG-3'	2968
		Dr.kcnh1b-R	5'-CGGCGCGCCCACCCTTTGAAGATATATTGTCATCCTTTTCTGAATCAGGCGAG-3'	
kcnh2a	NM_001042722.2	Dr.kcnh2a-F	5'-CGCCCCCTTCACCATGCCTGTACGACGGGGACACGTTGC-3'	3759
		Dr.kcnh2a-R	5'-CGGCGCGCCCACCCTTGAGCCAGGATCAGAGGGATGTCTTTTCTGCATTG-3'	
kcnh2b	XM_073941219.1	Dr.kcnh2b-F	5'-GCCCCCTTGCCACCATGCCGGTGCGAAGAGGACACGTCGC-3'	3278
		Dr.kcnh2b-R	5'-CGGCGCGCCCACCCTTGCTGCCGGGGTCTGAGCTGTGC-3'	
kcnh3	XM_001919401.8	Dr.kcnh3-F	5'-GCCCCCTTGCCACCATGCCTGTGATGAGAGGTCTGCTGGC-3'	3576
		Dr.kcnh3-R	5'-CGGCGCGCCCACCCTTCAGTGGTGGTCCTTCTTCATCTATGAAGCTG-3'	
kcnh4a	NM_001322433.1	Dr.kcnh4a-F	5'-GCCCCCTTGCCACCATGCCGGTGATGAAAGGGCTCCTGG-3'	3453
		Dr.kcnh4a-R	5'-CGGCGCGCCCACCCTTCTGAGCGACTGTGTTTTGCTCCTCAACAG-3'	
kcnh4b	XM_073918295.1	Dr.kcnh4b-F	5'-GCCCCCTTGCCACCATGCCAGTAATGAAGGGGCTGCTGGC-3'	3580
		Dr.kcnh4b-R	5'-CGGCGCGCCCACCCTTGTCTATTAGATCCAGGCACCAAGTGGTCTCTG-3'	
kcnh5a	NM_001276280.1	Dr.kcnh5a-F	5'-GCCCCCTTGCCACCATGCACGGGGGAAAAAGAGGACTGGTG-3'	3279
		Dr.kcnh5a-R	5'-CGGCGCGCCCACCCTTAAGCGGCCCATCATCGCCCTCCATC-3'	
kcnh5b	XM_002664207.7	Dr.kcnh5b-F	5'-GCCCCCTTGCCACCATGCCCGGGGGAAAGAGAGGGCTGG-3'	3015
		Dr.kcnh5b-R	5'-CGGCGCGCCCACCCTTTGGCAAGCCCTCATCTTTTTCAGAGTCCGG-3'	
kcnh6a	NM_212837.1	Dr.kcnh6a-F	5'-GCCCCCTTGCCACCATGCCCGTGCGCCGCGGACATGTC-3'	3555

		Dr.kcnh6a-R	5'-CGGCGCGCCCACCCTTGCTTCCGGGTAAGACTGGATCGGACACG-3'	
kcnh6b	XM_021472507.3	Dr.kcnh6b-F	5'-GCCCCCTTGCCACCATGCAGCGCTTCAACGGCTTGAAGAGC-3'	2679
		Dr.kcnh6b-R	5'-CGGCGCGCCCACCCTTGCTGACCGGTAATCCAGGATCAGACATGTG-3'	
kcnh7a	XM_073953689.1	Dr.kcnh7a-F	5'-GCCCCCTTGCCACCATGCCTGTGCGGAGAGGTCATGTC-3'	3405
		Dr.kcnh7a-R	5'-CGGCGCGCCCACCCTTGTGCTGCTGGTCGGGTGGTCC-3'	
kcnh7b	XM_073913232.1	Dr.kcnh7b-F	5'-GCCCCCTTGCCACCATGCCGGTGCGACGGGGTCACGTC-3'	3531
		Dr.kcnh7b-R	5'-CGGCGCGCCCACCCTTCTGCCCTGGAAGGCCGGGATCAGAG-3'	
kcnh8	XM_068215273.2	Dr.kcnh8-F	5'-GCCCCCTTGCCACCATGCCTGTTATGAAGGGATTGCTCGCTCCAC-3'	2988
		Dr.kcnh8-R	5'-CGGCGCGCCCACCCTTCAAACCTAGGGACCTGAGATCCTCCCCTTC-3'	
**Note: In-fusion cloning adapter sequences are derlined**				
